# Does preoperative dipyridamole-thallium scanning reduce 90-day cardiac complications and 1-year mortality in patients with femoral neck fractures undergoing hemiarthroplasty?

**DOI:** 10.1186/s13018-020-01918-w

**Published:** 2020-09-07

**Authors:** Chin-Yi Liao, Timothy L. Tan, Yu-Der Lu, Cheng-Ta Wu, Mel S. Lee, Feng-Chih Kuo

**Affiliations:** 1 Department of Orthopedic Surgery, Kaohsiung Chang Gung Memorial Hospital, College of Medicine, Chang Gung University, No. 123, Ta Pei Road, Niao Sung Dist, Kaohsiung, Taiwan; 2 Rothman Institute Orthopedic Research Department, Thomas Jefferson University, Rothman Institute Sheridan Building, Suite 1000, 25 S 9th Street, Philadelphia, PA 19107 USA

**Keywords:** Dipyridamole-thallium scanning, Femoral neck fracture, Mortality, Cardiac complications, Hemiarthroplasty

## Abstract

**Background:**

This study aimed to evaluate the effect of dipyridamole-thallium scanning (DTS) on the rates of 90-day cardiac complications and 1-year mortality in patients with a femoral neck fracture treated with hemiarthroplasty.

**Methods:**

Between 2008 and 2015, 844 consecutive patients who underwent cemented or cementless hemiarthroplasty were identified from the database of a single level-one medical center. One-hundred and thirteen patients (13%) underwent DTS prior to surgery, and 731 patients (87%) did not. Patient characteristics, comorbidities, surgical variables, and length of the delay until surgery were recorded. A propensity score-matched cohort was utilized to reduce recruitment bias in a 1:3 ratio of DTS group to control group, and multivariate logistic regression was performed to control confounding variables.

**Results:**

The incidence of 90-day cardiac complications was 19.5% in the DTS group and 15.6% in the control group (*p* = 0.343) among 452 patients after propensity score-matching. The 1-year mortality rate (10.6% vs 13.3%, *p* = 0.462) was similar in the two groups. In the propensity score-matched patients, utilization of DTS was not associated with a reduction in the rate of 90-day cardiac complications (matched cohort, adjusted odds ratio [aOR] = 1.32; 95% confidence interval [CI] 0.75–2.33, *p* = 0.332) or the 1-year mortality rate (aOR = 0.62; 95% CI 0.27–1.42, *p* = 0.259). Risk factors for cardiac complications included an American Society of Anesthesiologists grade ≥ 3 (OR 3.19, 95% CI 1.44–7.08, *p* = 0.004) and pre-existing cardiac comorbidities (OR 5.56, 95% CI 3.35–9.25, *p* < 0.001). Risk factors for 1-year mortality were a long time to surgery (aOR 1.15, 95% CI 1.06–1.25, *p* = 0.001), a greater age (aOR 1.05, 95% CI 1.00 to 1.10, *p* = 0.040), a low body mass index (BMI; aOR 0.89, 95% CI 0.81–0.98, *p* = 0.015), and the presence of renal disease (aOR 4.43, 95% CI 1.71–11.46, *p* = 0.002).

**Discussion:**

Preoperative DTS was not associated with reductions in the rates of 90-day cardiac complications or 1-year mortality in patients with a femoral neck fracture undergoing hemiarthroplasty. The necessity for DTS should be re-evaluated in elderly patients with femoral neck fractures, given that this increases the length of the delay until surgery.

**Level of evidence:**

Prognostic level III

## Introduction

Femoral neck fracture is a geriatric low-energy trauma with an increasing incidence in Asia [1] and has profound economic and medical impacts worldwide [2, 3]. The standard treatment for displaced femoral neck fractures in geriatric patients is arthroplasty [4]; however, patients may experience significant morbidity and mortality due to pre-existing cardiovascular comorbidities and insult from surgery [5]. Therefore, it is common practice to hold a preoperative consultation with internal medicine doctors or anesthesiologists for risk stratification and treatment optimization.

Dipyridamole-thallium scanning (DTS), or 201-scintigraphy, has been suggested as a method of preoperatively assessing cardiac risk in patients undergoing major non-cardiac surgery. The American College of Cardiology (ACC) and American Heart Association (AHA) guidelines include DTS as one of the modalities for perioperative cardiovascular evaluation prior to non-cardiac surgery [6, 7]. In terms of preoperative practice in other specialties, for instance, Landesberg et al. [8] examined the use of thallium scanning and selective coronary angiography prior to major vascular therapy and reported a significant improvement in the long-term outcome. Yin et al. [9] also demonstrated that applying preoperative DTS can prevent cardiac mortality and complications in patients undergoing parathyroidectomy. Hemiarthroplasty is classified as intermediate cardiac risk procedure, with reported perioperative morbidity rates ranging from 1 to 5% according to the ACC/AHA preoperative guidelines [6]. However, there is minimal literature on whether this examination results in any differences in outcomes in patients undergoing surgery for the treatment of a hip fracture.

The purpose of this study was to evaluate the correlations of DTS with the rates of 90-day cardiac complications and 1-year mortality in patients with a femoral neck fracture treated with hemiarthroplasty. We hypothesized that if DTS reduces the risk of postoperative complications, or if further selective preoperative treatment such as coronary intervention may be indicated in light of the results of DTS, the rate of postoperative cardiac complications may be reduced by balancing the bias in these patients.

## Materials and methods

### Data source and patients

The Institutional Review Board (IRB) approved the study protocol, and patient consent was waived by the IRB, as all personal identifying information was removed from the dataset and strict anonymity was maintained before further analysis. The duration of IRB approval was from August 2016 to October 2016, and privacy regulations related to the research database in our institution were adhered to in order to prevent confidential information being utilized against medical ethics. We retrospectively enrolled 968 consecutive patients undergoing hemiarthroplasty whose data were obtained from a trauma database held at a single academic institution from January 2008 to December 2015. Raw data acquisition was completed in 2 months; however, data completeness, reliability, organization details, and patient exclusions were manually reviewed by 3 independent authors (CYL, YDL, and CTW) to prevent observation bias. The inclusion criteria were patients with displaced femoral neck fractures who were older than 60 years. Patients with a non-displaced fracture, those aged under 60 years, and those with multiple trauma, valgus impacted fracture, pathologic fracture, or open fracture were excluded. After application of the exclusion criteria, 844 patients with displaced femoral neck fractures who underwent cemented or cementless hemiarthroplasty were included in this study.

All patients underwent a preoperative medical examination that included a resting electrocardiogram, chest radiography and blood test. Following this screening, they were seen by an anesthesiologist before surgery, and a cardiology consultation was obtained if concerns were raised by the anesthesiologist. The cardiologist or physician evaluated the patients undergoing non-cardiac surgery based on the 2007 ACC/AHA guidelines on perioperative cardiovascular evaluation [6]. As per the guidelines, routine screening with non-invasive stress testing was not performed in patients undergoing low-risk non-cardiac surgery; however, if the test results impacted on our decision-making, including canceling the operation or changing the perioperative care, this testing was arranged for patients with an elevated cardiac risk and a poor metabolic equivalent of task (MET < 4). If the patient had an elevated cardiac risk but a functional capacity greater than or equal to a MET of 4 without symptoms, the planned surgery would proceed.

Patients were divided into two categories based on receipt of DTS. The control group included patients who underwent surgery without DTS after consultation with an anesthesiologist or cardiologist, while the study group included patients who underwent DTS. Patients with abnormal scan results were further evaluated by a cardiologist with regard to coronary angiography intervention. Indications for coronary angiography were based on clinical findings, such as new or medically unstable angina, previous or recent myocardial infarction, or persistent angina. Stent implantation or balloon angioplasty during angiography was performed if coronary arterial stenosis was greater than 50%, multiple-vessel disease was present, or left main coronary artery occlusion was observed.

Variables of interest were extracted by electronic query of medical records and reviewed manually. The following information was obtained: basic demographic data, age, gender, body mass index (BMI), American Society of Anesthesiologists (ASA) grade, and preoperative comorbidities, including ischemic heart disease, congestive heart failure, valvular heart disease, cerebrovascular accident (CVA), diabetes, chronic obstructive pulmonary disease (COPD), rheumatoid arthritis, liver disease, renal disease, and a history of cancer (Table 1). In addition, the duration from hospital presentation to surgery*,* anesthesia type (regional or general), use of cemented or cementless fixation, duration of surgery, and need for transfusion were also obtained.

**Table 1:** Baseline demographic data, surgical factors, and length of stay in unmatched and matched patients who did and did not undergo a dipyridamole-thallium scan

		Non-thallium scan group			
	Thallium scan group (*n* = 113)	Unmatched cohort (*n* = 731)	*p* value	Matched cohort (*n* = 339)	*p* value
Age, mean ± SD, years	79.8 ± 8.3	74.7 ± 11.3	< 0.001	79.9 ± 8.3	0.901
Sex, *n* (%)			0.590		0.908
Male	37 (32.7)	221 (30.2)		109 (32.2)	
Female	76 (67.3)	510 (69.8)		230 (67.8)	
Body mass index, mean ± SD, kg/m^2^	22.6 ± 3.5	22.7 ± 4.1	0.774	22.5 ± 3.9	0.980
ASA grade, *n* (%)			< 0.001		0.010
1	0 (0.0)	11 (1.5)		2 (0.6)	
2	5 (4.4)	185 (25.3)		57 (16.8)	
3	106 (93.8)	523 (71.5)		270 (79.6)	
4	1 (0.9)	10 (1.4)		8 (2.4)	
Delay until surgery, mean ± SD, days	7.0 ± 12.4	2.4 ± 3.3	< 0.001	3.7 ± 4.2	< 0.001
Delay until surgery > 2 days, *n* (%)	110 (97.3)	352 (48.2)	< 0.001	127 (37.5)	< 0.001
Preoperative comorbidities, *n* (%)					
Ischemic heart disease	25 (22.1)	56 (7.7)	< 0.001	47 (13.9)	0.038
CHF	6 (5.3)	27 (3.7)	0.431	20 (5.9)	0.816
Valvular heart disease	11 (9.7)	18 (2.5)	0.001	17 (5.0)	0.071
CVA	11 (9.7)	76 (10.4)	0.829	34 (10.0)	0.928
Diabetes	44 (38.9)	216 (29.5)	0.044	123 (36.3)	0.613
COPD	13 (11.5)	53 (7.3)	0.117	31 (9.1)	0.464
RA	1 (0.9)	14 (1.9)	0.440	3 (0.9)	1.000
Liver disease	2 (1.8)	37 (5.1)	0.121	7 (2.1)	1.000
Renal disease	14 (12.4)	59 (8.1)	0.129	37 (10.9)	0.668
Cancer	4 (3.5)	48 (6.6)	0.213	12 (3.5)	1.000
Anesthesia type, *n* (%)			0.071		0.597
General	94 (83.2)	651 (89.1)		289 (85.3)	
Regional	19 (16.8)	80 (10.9)		50 (14.7)	
Cemented stem, *n* (%)	65 (57.5)	297 (40.6)	< 0.001	199 (58.7)	0.826
Time in surgical theater, mean ± SD, min	125.9 ± 36.3	132.3 ± 44.5	0.142	125.5 ± 33.8	0.930

*ASA* American Society of Anesthesiologists, *CHF* congestive heart failure, *CVA* cerebrovascular accident, *COPD* chronic obstructive pulmonary disease, *RA* rheumatoid arthritis, *SD* standard deviation

### Surgical techniques

All hemiarthroplasty surgeries were performed through the posterolateral approach in a lateral decubitus position. All patients were treated using a cemented or cementless fixation technique by well-trained orthopedic surgeons. The cemented or press-fit implants were chosen based on preoperative imaging evaluation for osteoporosis and intraoperative trial implantation at the discretion of the treating surgeons. Intraoperative joint capsules were approximated by sutures, and a closed drainage tube was inserted, which was removed within 24–48 h. A partial weight-bearing rehabilitation program was initiated by a physical therapist from postoperative day 1. If a patient was unable to ambulate using a walker, wheelchair ambulation was recommended.

### Outcome measurement

The outcomes of this study included the rates of postoperative 90-day cardiac complications, 90-day mortality, and 1-year mortality after the index hemiarthroplasty. ICD-9 codes were used to identify 90-day cardiac complications, including decompensated heart failure, arrhythmia, sequelae of coronary artery disease, angina pectoris, or acute myocardial infarction, while mortality was defined as any death after the surgery. The mortality data were obtained from the Health and Welfare Data Science Center, Ministry of Health and Welfare (HWDC, MOHW), who hold a set of healthcare-related databases that provide information from over 30 departments and ministries, including the National Health Insurance Research Database (NHIRD). The NHIRD is an administrative dataset derived from Taiwan’s universal National Health Insurance program and contains records of the enrolled 99% of the Taiwanese population.

### Statistical analysis

All variables were tested for normality using the Kolmogorov-Smirnov test. Student’s *t* test was utilized for normally distributed continuous variables, and the Mann-Whitney *U* test was used for nonparametric data. Fisher’s exact test or the chi-squared test was used for categorical variables. Comparison was performed before and after applying propensity score-matching.

Propensity score-matched analysis uses a propensity score calculated from covariate data to match patients from different study groups. The technique is often applied to eliminate selection bias in orthopedic observational studies [10]. We employed this technique (1:3 ratio of DTS group to control group) with a logistic regression model to adjust the covariates, including basic demographic data (age, gender, BMI, and ASA grade), preoperative comorbidities (all cardiac comorbidities, CVA, diabetes, COPD, rheumatoid disease, liver disease, renal disease, and cancer history), surgical variables (anesthesia type, use of a cemented or cementless stem, duration of surgery, and transfusion), and time interval from hospital admission to surgery, in order to decrease the differences in baseline conditions between groups and to minimize selection bias in the performance of DTS. Univariate and multivariate logistic regression analyses were also conducted to determine whether DTS was an independent factor affecting the rates of 90-day cardiac complications and 1-year mortality after adjusting for the confounding factors listed above. Odds ratios (ORs) with 95% confidence intervals (CIs) were calculated, and a *p* value of less than 0.05 was taken to indicate statistical significance. All statistical analyses were performed using Statistical Package for Social Science (SPSS) version 22 software (SPSS Inc., IBM, Armonk, NY, USA) and NCSS Statistical Analysis and Graphics software (NCSS, LLC, Kaysville, UT, USA).

## Results

There were several baseline differences between the patients who underwent preoperative DTS and those who did not before propensity score-matching. The patients who underwent DTS were of an older age (*p* < 0.001) and a higher ASA grade (*p* < 0.001), and higher proportions of those patients had ischemic heart disease (22.1% vs 7.7%, *p* = 0.001), valvular heart disease (9.7% vs 2.5%, *p* = 0.001), and diabetes mellitus (38.9% vs 29.5%, *p* = 0.044). In addition, cemented hemiarthroplasty was more common in these patients (57.5% vs. 40.6%, *p* < 0.001). Furthermore, patients who underwent DTS had an average wait of 4.6 days longer to receive surgery (7.0 ± 12.4 days vs. 2.4 ± 3.3 days, *p* < 0.001).

After performing propensity score-matching, patients who underwent DTS had a higher ASA grade (*p* = 0.01), and a higher proportion of those patients had ischemic heart disease (*p* = 0.038). Further standardized difference analysis of ASA grade showed a reduction from 62.8% to 32.1%, while the standardized difference of ischemic heart disease was reduced from 36.0 to 18.0%. All other covariates and comorbidities were statistically balanced after each examination of the Student *t* test and chi-square test results. Patients who underwent DTS still had a longer preoperative delay until surgery (7.0 ± 12.4 days vs. 3.7 ± 4.2 days, *p* < 0.001).

In our study, the 90-day cardiac complications observed were as follows: 2 patients (0.2%) with angina pectoris; 32 (3.7%) with symptomatic heart failure; 60 (7.1%) with cardiac arrhythmia, including 53 with atrial fibrillation with rapid ventricular response, 5 with right bundle branch block, and 2 with episodic paroxysmal supraventricular tachycardia (asymptomatic sinus bradycardia or tachycardia; first-degree atrioventricular block was excluded); 1 (0.1%) with a history of thoracic aortic dissection; and 6 (0.7%) with acute myocardial infarction. After propensity score-matching, the incidence of 90-day cardiac complications was 19.5% in the DTS group and 15.6% in the control group (*p* = 0.343).

**Fig. 1:**
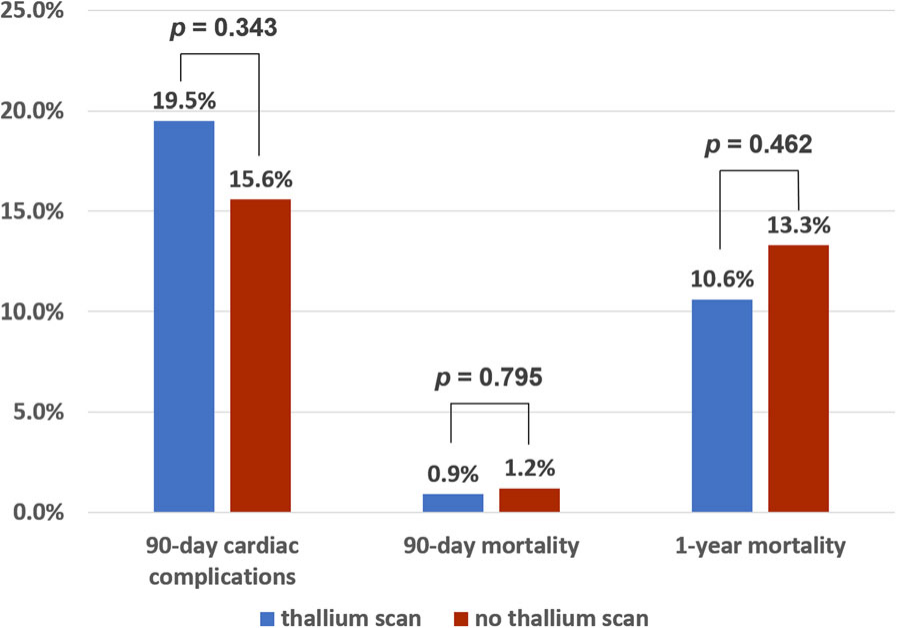
Rates of 90-day cardiac complications, 90-day mortality, and 1-year mortality in patients who did and did not undergo a dipyridamole-thallium scan after propensity score-matching

The 90-day mortality (0.9% vs. 1.2%, *p* = 0.795) and 1-year mortality rates (10.6% vs 13.3%, *p* = 0.462) were similar between the two groups (Fig. 1). Furthermore, we regarded the 23 patients with positive DTS findings as a subgroup and compared this group with the remaining 90 patients with negative DTS findings and the 339 patients in the control group. The 1-year mortality rate was still not significantly different between the three subgroups according to the results of the chi-square test (*p* = 0.706).

The correlations of DTS with the rates of 90-day cardiac complications, 90-day mortality, and 1-year mortality are depicted in Table 2. Multivariate analysis revealed no significant associations of DTS with the rates of 90-day cardiac complications (adjusted OR [aOR] = 1.44; 95% CI 0.71 to 2.91, *p* = 0.309), 90-day mortality (aOR = 9.80; 95% CI 0.23 to 424.80, *p* = 0.235), or 1-year mortality (aOR = 0.54 95% CI 0.24 to 1.20, *p* = 0.128). Analysis of the propensity score-matched cohort also confirmed no significant differences in terms of the correlations of DTS with the rates of 90-day cardiac complications (aOR = 1.32; 95% CI 0.75 to 2.33, *p* = 0.332), 90-day mortality (aOR = 0.75; 95% CI 0.08 to 6.71, *p* = 0.797), or 1-year mortality (aOR = 0.622, 95% CI 0.27 to 1.42, *p* = 0.259). We performed further subgroup evaluation of cardiac complications in order to assess the correlations of DTS with postoperative unstable angina and acute myocardial infarction, and no statistically significant differences between the DTS group and the non-DTS group were observed (*p* = 0.437 and 0.272, respectively).

**Table 2:** Univariate and multivariate logistic regression models evaluating the associations of dipyridamole-thallium scan with the rates of 90-day cardiac complications, 90-day mortality, and 1-year mortality

Outcome	Unmatched cohort				Matched cohort	
	Unadjusted OR (95% CI)	*p* value	Adjusted OR^a^ (95% CI)	*p* value	Adjusted OR^b^ (95% CI)	*p* value
Overall 90-day cardiac complications	1.86 (1.11–3.13)	0.019	1.44 (0.71–2.91)	0.309	1.32 (0.75–2.33)	0.332
Unstable angina	6.52 (0.41–104)	0.186	4.26 (0.12–154)	0.429	3.02 (0.19–48.1)	0.437
Acute myocardial infarction	3.28 (0.59–18.1)	0.174	2.83 (0.38–21.2)	0.310	3.04 (0.42–21.3)	0.272
90-day mortality	0.92 (0.11–7.58)	0.941	9.80 (0.23–424.8)	0.235	0.75 (0.08–6.71)	0.797
1-year mortality	1.09 (0.57–2.08)	0.799	0.54 (0.24–1.20)	0.128	0.62 (0.27–1.42)	0.259

^a^Adjusted for age, sex, body mass index, American Society of Anesthesiologists (ASA) grade, preoperative comorbidities (ischemic heart disease, congestive heart failure, valvular heart disease, cerebrovascular accident, diabetes, chronic obstructive pulmonary disease, rheumatoid arthritis, liver disease, renal disease, and cancer history), surgical variables (anesthesia and cemented stem, operative duration, and transfusion), and duration to surgery

^b^Adjusted for ASA grade and ischemic heart disease

*OR* odds ratio, *CI* confidence interval

Independent risk factors for postoperative 90-day cardiac complications included an ASA grade ≥ 3 (OR 3.19, 95% CI 1.44 to 7.08, *p* = 0.004) and pre-existing cardiac comorbidity (OR 5.56, 95% CI 3.35 to 9.25, *p* < 0.001) in the multivariate analysis (Table 3). Independent risk factors for 1-year mortality were a long delay until surgery (OR 1.15, 95% CI 1.06 to 1.25, *p* = 0.001), an older age (OR 1.05, 95% CI 1.00 to 1.10, *p* = 0.040), a low BMI (OR 0.89, 95% CI 0.81 to 0.98, *p* = 0.015), and the presence of renal disease (OR 4.43, 95% CI 1.71 to 11.46, *p* = 0.002) in the multivariate analysis (Table 4).

**Table 3:** Results of multivariate logistic regression regarding 90-day cardiac complications

	Beta coefficient	Standard error	OR	95% CI	*p* value
ASA grade ≥ 3	1.16	0.41	3.19	1.44 to 7.08	0.004
Cardiac comorbidities	1.72	0.26	5.56	3.35 to 9.25	< 0.001

Cardiac comorbidities included ischemic heart disease, congestive heart failure, and valvular heart disease

*OR* odds ratio, *CI* confidence interval, *ASA* American Society of Anesthesiologists

**Table 4:** Results of multivariate logistic regression regarding 1-year mortality

	Beta coefficient	Standard error	OR	95% CI	*p* value
Delay until surgery, days	0.14	0.42	1.15	1.06 to 1.25	0.001
Age, years	0.05	0.02	1.05	1.00 to 1.10	0.040
Body mass index, kg/m^2^	-0.11	0.05	0.89	0.81 to 0.98	0.015
Renal disease	1.49	0.49	4.43	1.71 to 11.46	0.002

*OR* odds ratio, *CI* confidence interval

**Fig. 2:**
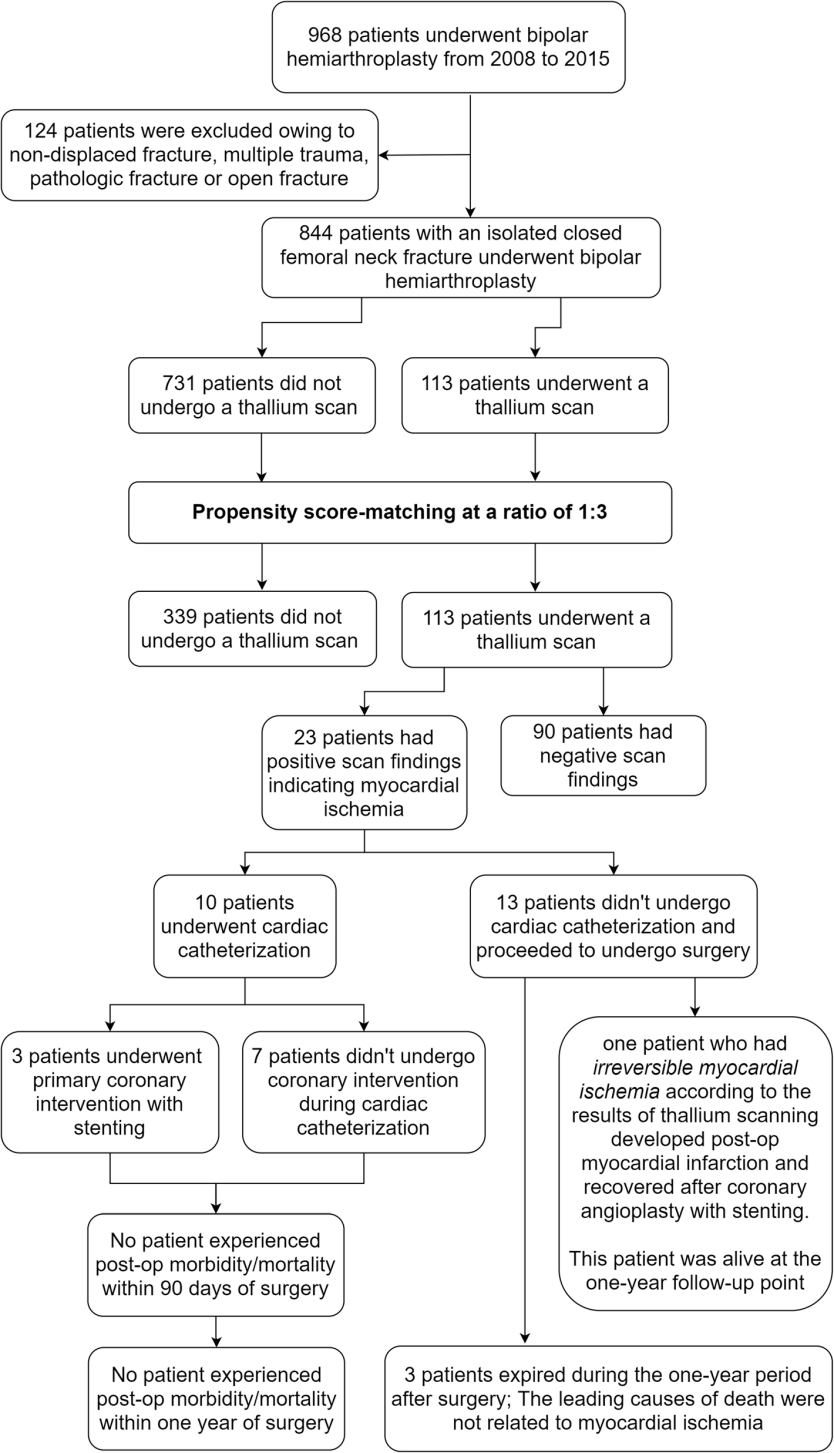
Flowchart showing the selection process of patients and the further decision-making in the two groups, categorized according to performance of a dipyridamole-thallium scan

Of the 113 patients who underwent DTS, 90 (79.7%) had negative findings and 23 (20.3%) were found to have reversible myocardial ischemia. According to the follow-up records of these 23 patients, 10 (8.8%) underwent cardiac catheterization, but only 3 (2.7%) underwent a primary coronary intervention (PCI) with stenting, using either bare-metal or drug-eluting stents, or balloon angioplasty; the 7 patients (6.2%) who did not undergo a PCI during cardiac catheterization were treated in accordance with the judgment of a cardiologist. All of the 10 patients who underwent cardiac catheterization or further PCI received surgery eventually, and none of these 10 patients had postoperative cardiac complications or died within 90 days. Of the 13 patients (11.5%) who underwent DTS and who did not undergo cardiac catheterization, one developed postoperative myocardial infarction, but soon recovered after PCI with stenting; the follow-up records of this patient for 1 postoperative year were examined, and no further cardiac morbidity or mortality was documented (Fig. 2).

All of the 23 patients in the DTS group with reversible myocardial ischemia were alive at 90 days postoperatively. Three patients (13%) who did not receive a cardiac catheterization were dead within 1 year after surgery. One of those patients had probable reversible myocardial ischemia, and the leading cause of death was respiratory failure after a benzodiazepine overdose. The second patient had three-vessel coronary artery disease, atrial fibrillation and a history of decompensated heart failure post-mechanical ventilation 1 year prior to surgery, and the leading cause of death was decompensated heart failure. There was no evidence of new onset of myocardial ischemia in the second patient according to the documented levels of cardiac enzymes and the results of electrocardiography. The final patient had an equivocal result in the thallium test, and the leading cause of death was pneumonia. In no case of mortality was there significant evidence of a relationship with myocardial ischemia.

## Discussion

The results of our study suggested that the rate of 90-day cardiac complications was still high despite the use of DTS (19.5% vs. 15.6%, Fig. 1). Utilization of preoperative DTS was not significantly associated with a change in the rate of 90-day overall cardiac complications (OR = 1.32; 95% CI 0.75 to 2.33; *p* = 0.332, Table 2) after controlling the confounders. Further subgroup evaluation of patients with postoperative unstable angina and acute myocardial infarction was performed, which also showed no statistically significant differences between the two groups (*p* = 0.437 and 0.272, respectively). We found that patients with an ASA grade ≥ 3 and those with pre-existing cardiac comorbidities (ischemic heart disease, congestive heart failure, and valvular heart disease) were associated with an increased risk of 90-day cardiac complications. Furthermore, among the patients who underwent DTS, only one (4.3%) had myocardial infarction after surgery, and no mortality was observed within 90 days postoperatively. We examined the records of the 23 patients with positive DTS findings for up to 1 year post-surgery; of these 23 patients, 3 who did not undergo cardiac catheterization were dead, but the leading causes were not directly related to myocardial ischemia. Our results demonstrated that the performance of DTS was not associated with a reduction in the rate of cardiac complications.

DTS was not found to be associated with a change in the 90-day mortality rate (aOR = 0.75; 95% CI 0.08 to 6.71; *p* = 0.797) or the 1-year mortality rate (aOR = 0.62, 95% CI 0.27 to 1.42; *p* = 0.259) (Table 2) after controlling the confounders in this study. We assumed that DTS may lead to fewer cardiac complications and alter the mortality rate in light of potential improvement in the selection of the most suitable coronary interventions after DTS. However, the mortality rate in the patients with positive DTS findings was not statistically significantly different to that in the patients with negative DTS findings or the control group (*p* = 0.706). The benefit of DTS was not as strong as has been demonstrated in studies examining other specialties, such as thyroid and cardiovascular surgery [8, 9].

A delay in performing surgery by 48 h was not an independent factor related to the rate of 90-day complications according to the results of multivariate logistic regression (OR1.50, 95% CI 0.76 to 2.95, *p* = 0.243); however, it was significantly related to the rate of 90-day cardiac complications according to Pearson chi-square analysis (*p* = 0.035). These results may imply that delayed surgery did not affect patient outcomes directly but was a reflection of the comorbidities that contributed to postoperative complications. Thus, in patients with cardiac comorbidities or an ASA score of 3 or 4, surgery could have been delayed in order to allow improvement in the general condition of the patient. It is difficult to set precise cut-off points to define early and delayed surgery, but generally, the optimal timing might be considered between 12 and 48 h after presentation at the hospital based on current evidence [11].

It is worth mentioning that a long delay until surgery (OR 1.15, 95% CI 1.06 to 1.25, *p* = 0.001) was associated with the 1-year mortality rate in our study. Several previous studies found that a prolonged duration until surgery (≥ 48 h) for hip fractures may increase the 30-day and 1-year mortality rates [12, 13]. However, it is a frequent practice that preoperative consultation with another service is obtained for risk stratification and medical optimization. While the goal is to make a decision to proceed immediately or medically optimize the patient first, the process of preoperative medical evaluation usually takes 1 to 2 days before the patient can be taken to surgery [14]. Among the common preoperative examinations, DTS may take longer to organize, as it is often dependent on scanner availability and time of day [15–18], and an average delay of 4 days was observed at our institution.

Being overweight is generally considered a risk factor related to high morbidity and mortality, but the results of recent studies suggested that obese patients may have had a lower mortality rate after hip fracture in old patients with comorbidities. This phenomenon is termed the “obesity paradox” in the literature [19], but there is debate over the evidence and physiology. Hedstrom et al. [20] assumed that there exists a prolonged catabolic response after stress suffered following a hip fracture, and patients with a higher BMI may have a greater metabolic reserve to enable them to better endure this stress, which was also found to be the case in critical illness [21]. The results of our study suggested a higher 1-year mortality rate in patients of low BMI, which was compatible with previous literature (OR 0.89, 95% CI 0.81 to 0.98, *p* = 0.015), and this indicates the importance of nutritional support, especially in patients with cachexia.

The incidence of hip fracture is high among patients with renal failure due to osteoporosis and osteodystrophy, and there is evidence to suggest that patients with chronic kidney disease (CKD) have a higher mortality rate after hip fracture in the elderly [22]. Postoperative care of CKD patients is always challenging due to difficulty in fluid management, higher comorbidity, and catastrophic complications after acute exacerbation of renal failure. Our research indicated a higher 1-year mortality rate in patients with renal disease (OR 4.43, 95% CI 1.71 to 11.46, *p* = 0.002), and a multi-discipline protocol should be established in the future, as these patients may not participate in standard rehabilitation and osteoporosis treatment.

Ricci et al. [23] demonstrated that preoperative cardiac testing (DTS, echocardiography, and cardiac catheterization) did not change the management of perioperative orthopedic surgery or medical therapy in elderly patients with hip fractures but did incur a huge cost of over $47 million annually in the USA; patients undergoing cardiac testing had a delay to surgery on average 3.3 days, which was 1.4 days greater than those who did not. Multiple studies have also demonstrated that preoperative cardiac testing with echocardiography delays surgery without a significant change in preoperative cardiac medications or anesthesia [14, 24–26]. In addition, the cost of a thallium test is 261.22 dollars at our institution. Compared with previous studies, we included more patients in our study; moreover, we performed propensity score analysis to control possible group differences in patients’ background conditions and reduce the effect of selection bias. We also analyzed the independent factors related to postoperative cardiac complications and the 1-year mortality rate. It is worth mentioning that we extracted mortality data from the records of the Health and Welfare Data Science Center, which included over 30 medical institutions and over 99% of the population of Taiwan. Accordingly, our mortality data were reliable, and this strengthened the power of the analysis.

It is rational to consider that perioperative cardiac assessment could lead to safer surgery and fewer cardiac complications. However, the results of previous studies did not indicate increased benefit in cases of geriatric hip trauma. Thus, we suppose that over-screening may occur in daily practice. Currently, during preoperative assessment by a cardiologist following the ACC/AHA guidelines, patients who have a functional capacity of < 4 MET require further evaluation, which is arbitrary and subjective and may lead to over-diagnosis and incorrect indications of cardiac examinations. Vigoda et al. [27] performed a study of 548 anesthesiologist residents nationwide and examined their judgment in different scenarios. Fewer than half of the participants adhered to correct practice in accordance with standard care as set down in the 2007 ACC/AHA guidelines. We also assumed that the evolving minimally invasive technique of hemiarthroplasty with a reduced surgical duration, in conjunction with postoperative early rehabilitation with mechanical/pharmaceutic thromboembolism prophylaxis, would result in a lower incidence of postoperative morbidity as compared with the same surgery decades ago. There may exist an imbalance between performance of an expensive nuclear imaging test, which delays surgery for up to 4 days, and its clinical benefit.

Highly selective coronary angiography and interventions are no doubt helpful in terms of decreasing postoperative morbidity/mortality. In our study, 113 patients underwent DTS, but only 10 received coronary angiography, and only 3 of those 10 patients required interventions with a stenting procedure (2.7%). Thus, we identified a low rate of selective angiography with PCI after obtaining the results of DTS, which may account for a decreased clinical benefit of testing. The thallium scan has a questionable negative predictive value in patients with “balanced ischemia” due to global myocardial ischemia leading to poor interpretation of a relative perfusion defect in affected segments, which is a common phenomenon in nuclear imaging [28–30].

It is necessary to develop a better cardiac assessment tool with greater specificity that is less time-consuming to perform and has a better negative predictive value. Focused transthoracic echocardiography, or goal-directed focused cardiac ultrasound (FoCUS), has garnered interest for its use in perioperative assessment under specific conditions, especially for urgent surgery, due to its availability, portability, and time-saving property [31, 32]. Canty et al. [33] performed a prospective observational study of 99 patients who had suspected cardiac disease or were older than 65 years, in which preoperative focused echocardiography was performed by an anesthesiologist. In that study, this examination allowed identification of 64% of patients with cardiac disease, and clinical measures including referral to a cardiologist, change to the type of surgery performed, or intraoperative optimization were implemented in 36% of patients. However, there has been no comparative study of formal stress echocardiography and bedside FoCUS, and there remains uncertainty around the appropriate level of experience required in order to decrease the number of false negatives. Currently, there is no consensus with regard to a thallium scan substitute, but computed tomographic angiography (coronary CTA) has been noted to be useful. Huang et al. [34] investigated the value of coronary CTA in non-cardiac surgeries and found that this examination yielded improved perioperative risk stratification with a low rate of major cardiac events, high specificity, and good negative predictive value. The risk of a major cardiac event was reported to be 14% in patients with significant CTA findings [34]. Future research involving prospective, randomized allocation of patients is warranted to minimize selection bias and group differences.

There were several limitations of this study that should be considered. Most notably, there may have been selection bias, as DTS was performed in high-risk patients who were likely inherently subject to a higher risk of 90-day cardiac complications. We attempted to balance the baseline patient condition and control for potential confounders, but the DTS group still had a higher ASA grade, higher rates of ischemic heart disease and delayed surgery, even further standardized difference analysis showed reduction of imbalance between groups. Second, the decision to utilize DTS was made by different cardiologists, and thus there may be some variation in patient stratification and the cardiologists’ willingness to order additional cardiac tests. Third, we focused on the rates of 90-day cardiac morbidity and 1-year mortality and thus did not account for any complications that may have occurred beyond that follow-up duration.

## Conclusion

Preoperative DTS was not associated with reductions in the rates of 90-day cardiac complications and 1-year mortality in patients with a femoral neck fracture undergoing cemented or cementless hemiarthroplasty. The patients in whom DTS was performed underwent surgery 4 days later on average than the patients who did not, with no significant clinical benefit in terms of the rates of 90-day cardiac complications and 1-year mortality. Given the results of this study and those reported in existing literature, we propose that routine DTS in patients with an isolated femoral neck fracture should not be performed. We instead suggest that it is important to consider the patient’s preoperative cardiovascular comorbidity, underlying disease, and functional status during the preoperative assessment.

## Data Availability

Data are available from the corresponding author.

## Abbreviations

DTS: Dipyridamole-thallium scanning
AHA: American Heart Association
ASA: American Society of Anesthesiologists
CHF: Congestive heart failure
CVA: Cerebrovascular accident
COPD: Chronic obstructive pulmonary disease
RA: Rheumatoid arthritis
SD: Standard deviation
OR: Odds ratio
CI: Confidence interval

## References

[1] Cheung C-L, SBin A, Chadha M, Chow ES-L, Chung Y-S, Hew FL (2018). An updated hip fracture projection in Asia: the Asian Federation of Osteoporosis Societies study. Osteoporos Sarcopenia.

[2] Wu TY, Hu HY, Lin SY, Chie WC, Yang RS, Liaw CK (2017). Trends in hip fracture rates in Taiwan: a nationwide study from 1996 to 2010. Osteoporos Int..

[3] Kanis JA, Odén A, McCloskey EV, Johansson H, Wahl DA, Cooper C (2012). A systematic review of hip fracture incidence and probability of fracture worldwide. Osteoporos Int..

[4] Zielinski SM, Meeuwis MA, Heetveld MJ, Verhofstad MHJ, Roukema GR, Patka P (2013). Adherence to a femoral neck fracture treatment guideline. Int Orthop..

[5] D’Angelo F, Giudici M, Molina M, Margaria G (2005). Mortality rate after hip hemiarthroplasty: analysis of risk factors in 299 consecutives cases. J Orthop Traumatol..

[6] Fleisher LA, Beckman JA, Brown KA, Calkins H, Chaikof EL, Fleischmann KE (2007). ACC/AHA 2007 guidelines on perioperative cardiovascular evaluation and care for noncardiac surgery: a report of the American College of Cardiology/American Heart Association Task Force on Practice Guidelines Writing Committee to Revise the 2002 Guideline. Circulation.

[7] Fleisher LA, Fleischmann KE, Auerbach AD, Barnason SA, Beckman JA, Bozkurt B (2014). 2014 ACC/AHA guideline on perioperative cardiovascular evaluation and management of patients undergoing noncardiac surgery: a report of the American College of Cardiology/American Heart Association Task Force on Practice Guidelines. Circulation..

[8] Landesberg G, Mosseri M, Wolf YG, Bocher M, Basevitch A, Rudis E (2003). Preoperative thallium scanning, selective coronary revascularization, and long-term survival after major vascular surgery. Circulation..

[9] Yin S, Chou F-F, Wu S-C, Chi S-Y. Applying preoperative dipyridamole thallium-201 scintigraphy for preventing cardiac mortality and complications for patients with secondary hyperparathyroidism undergoing parathyroidectomy. Asian J Surg. 2017. 10.1016/j.asjsur.2017.03.004. 10.1016/j.asjsur.2017.03.004 28689732

[10] Inacio MCS, Chen Y, Paxton EW, Namba RS, Kurtz SM, Cafri G (2015). Statistics in brief: an introduction to the use of propensity scores. Clin Orthop Relat Res..

[11] Moja L, Piatti A, Pecoraro V, Ricci C, Virgili G, Salanti G (2012). Timing matters in hip fracture surgery : patients operated within 48 hours have better outcomes . A meta-analysis and meta-regression of over 190 , 000 patients.

[12] Shiga T, Wajima Z, Ohe Y (2008). Is operative delay associated with increased mortality of hip fracture patients? Systematic review, meta-analysis, and meta-regression. Can J Anesth Can d’anesthésie.

[13] Cluett J, Caplan J, Yu W (2008). Preoperative cardiac evaluation of patients with acute hip fracture. Am J Orthop (Belle Mead NJ).

[14] Marcantonio A, Steen B, Kain M, Bramlett KJ, Tilzey JF, Iorio R (2015). The clinical and economic impact of preoperative transthoracic echocardiography in elderly patients with hip fractures. Bull Hosp Jt Dis.

[15] Simunovic N, Devereaux PJ, Bhandari M (2011). Surgery for hip fractures: does surgical delay affect outcomes?. Indian J Orthop.

[16] Middleton RG, Uzoigwe CE, Young PS, Smith R, Gosal HS, Holt G (2014). Peri-operative mortality after hemiarthroplasty for fracture of the hip. Bone Joint J.

[17] Rodriguez-Fernandez P, Adarraga-Cansino D, Carpintero P (2011). Effects of delayed hip fracture surgery on mortality and morbidity in elderly patients. Clin Orthop Relat Res..

[18] Sheehan KJ, Sobolev B, Guy P, Kuramoto L, Morin SN, Sutherland JM (2016). In-hospital mortality after hip fracture by treatment setting. CMAJ..

[19] Modig K, Erdefelt A, Mellner C, Cederholm T, Talbäck M, Hedström M (2019). Obesity paradox holds true for patients with hip fracture: a registry-based cohort study. J Bone Jt Surg Am.

[20] Hedström M, Ljungqvist O, Cederholm T. Metabolism and catabolism in hip fracture patients: nutritional and anabolic intervention - a review. Acta Orthop. 2006;77:741–7. 10.1080/17453670610012926 17068704

[21] Hanna JS (2015). Sarcopenia and critical illness: a deadly combination in the elderly. J Parenter Enter Nutr..

[22] Yoon BH, Koo KH (2017). Hip fracture in chronic kidney disease patients: necessity of multidisciplinary approach. J Korean Med Sci..

[23] Ricci WM, GJDella R, Combs C, Borrelli J (2007). The medical and economic impact of preoperative cardiac testing in elderly patients with hip fractures. Injury.

[24] Sawhney C, Trikha V, Janani S, Bajwa SS, Sharma V, Khanna M (2017). Impact of preoperative echocardiography on perioperative management in geriatric hip trauma: a retrospective observational study. Int J Appl Basic Med Res..

[25] Mutlu H, Bilgili F, Mutlu S, Karaman O, Cakal B, Ozkaya U (2016). The effects of preoperative non-invasive cardiac tests on delay to surgery and subsequent mortality in elderly patients with hip fracture. J Back Musculoskelet Rehabil..

[26] OʼhEireamhoin S, Beyer T, Ahmed M, Mulhall KJ. (2011). The role of preoperative cardiac investigation in emergency hip surgery. J Trauma Inj Infect Crit Care..

[27] Vigoda MM, Sweitzer B, Miljkovic N, Arheart KL, Messinger S, Candiotti K (2011). 2007 American College of Cardiology/American Heart Association (ACC/AHA) guidelines on perioperative cardiac evaluation are usually incorrectly applied by anesthesiology residents evaluating simulated patients. Anesth Analg..

[28] Fathala A (2011). Myocardial perfusion scintigraphy: techniques, interpretation, indications and reporting. Ann Saudi Med..

[29] Heinle I, Siraj QH (2009). Artefacts and pitfalls in myocardial perfusion imaging. Integr Cardiol Nucl Med Physicians A Guid to Nucl Med Physicians.

[30] Aziz EF, Javed F, Alviar CL, Herzog E (2011). Triple vessel coronary artery disease presenting as a markedly positive stress electrocardiographic test and a negative SPECT-TL scintigram: a case of balanced Ischemia. Heart Int..

[31] Shim CY (2017). Preoperative cardiac evaluation with transthoracic echocardiography before non-cardiac surgery. Korean J Anesthesiol..

[32] Lenk T, Whittle J, Miller TE, Williams DGA, Bronshteyn YS (2019). Focused cardiac ultrasound in preoperative assessment: the perioperative provider’s new stethoscope?. Perioper Med..

[33] Canty DJ, Royse CF, Kilpatrick D, Williams DL, Royse AG (2012). The impact of pre-operative focused transthoracic echocardiography in emergency non-cardiac surgery patients with known or risk of cardiac disease. Anaesthesia..

[34] Hwang J, Kim E-K, Yang J-H, Chang S-A, YBin S, Hahn J-Y (2015). Assessment of perioperative cardiac risk of patients undergoing noncardiac surgery using coronary computed tomographic angiography. Circ Cardiovasc Imaging.

